# Advances in Nucleotide Repeat Expansion Diseases: Transcription Gets in Phase

**DOI:** 10.3390/cells12060826

**Published:** 2023-03-07

**Authors:** Ana S. Figueiredo, Joana R. Loureiro, Sandra Macedo-Ribeiro, Isabel Silveira

**Affiliations:** 1 Instituto de Investigação e Inovação em Saúde (i3S), Universidade do Porto, 4200-135 Porto, Portugal; 2 Instituto de Biologia Molecular e Celular (IBMC), Universidade do Porto, 4200-135 Porto, Portugal; 3 Instituto de Ciências Biomédicas Abel Salazar (ICBAS), Universidade do Porto, 4050-313 Porto, Portugal

**Keywords:** liquid/liquid phase separation, RNA-binding protein, RNA/protein aggregates, polyalanine, polyglutamine, NIID, spinocerebellar ataxia, frontotemporal dementia/amyotrophic lateral sclerosis, SCA37, FAME1

## Abstract

Unstable DNA repeat expansions and insertions have been found to cause more than 50 neurodevelopmental, neurodegenerative, and neuromuscular disorders. One of the main hallmarks of repeat expansion diseases is the formation of abnormal RNA or protein aggregates in the neuronal cells of affected individuals. Recent evidence indicates that alterations of the dynamic or material properties of biomolecular condensates assembled by liquid/liquid phase separation are critical for the formation of these aggregates. This is a thermodynamically-driven and reversible local phenomenon that condenses macromolecules into liquid-like compartments responsible for compartmentalizing molecules required for vital cellular processes. Disease-associated repeat expansions modulate the phase separation properties of RNAs and proteins, interfering with the composition and/or the material properties of biomolecular condensates and resulting in the formation of abnormal aggregates. Since several repeat expansions have arisen in genes encoding crucial players in transcription, this raises the hypothesis that wide gene expression dysregulation is common to multiple repeat expansion diseases. This review will cover the impact of these mutations in the formation of aberrant aggregates and how they modify gene transcription.

## 1. Introduction

**Figure 1:**
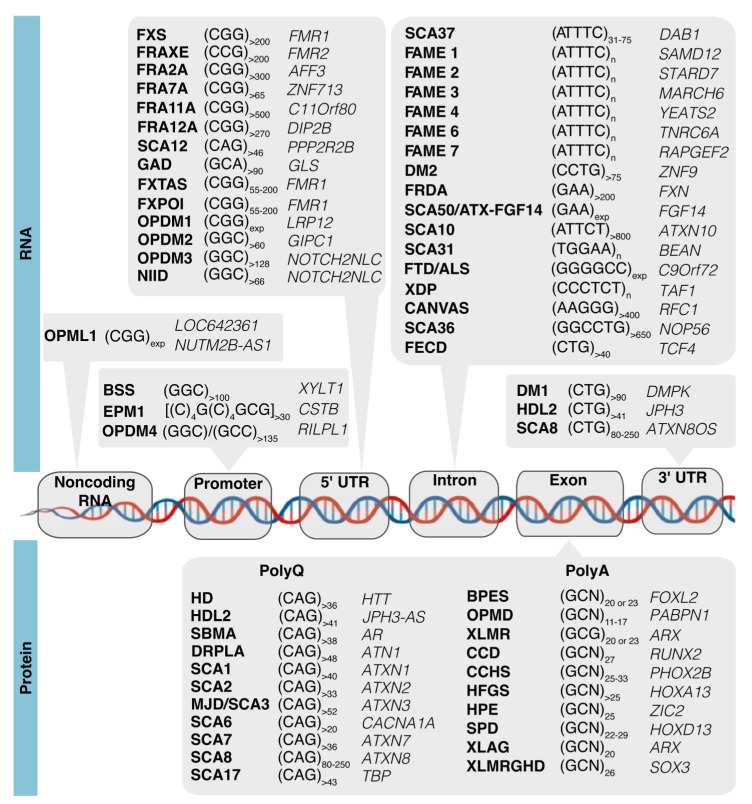
Disease-associated STR expansions can be located at coding or noncoding gene regions. In noncoding gene regions, STR expansions can be located at promoters such as in Baratella Scott syndrome (BSS) and oculopharyngodistal myopathy 4 (OPDM4); in 5′-UTR such as in FXS, fragile-X-associated tremor/ataxia syndrome (FXTAS), SCA12, neuronal intranuclear inclusion disease (NIID), and OPDM1–3; in 3′-UTR (e.g., DM1, HDL2 and SCA8) or introns (e.g., FTD/ALS, SCA31, SCA37, and SCA50); and in noncoding RNAs as in oculopharyngeal myopathy with leukoencephalopathy 1 (OPML1). In coding gene regions, the expansion of STRs result in expanded polyglutamine (polyQ) sequences in HD; SCA1–3, 6–8, and 17; or polyalanine (polyA) tracts in blepharophimosis syndrome (BPES), oculopharyngeal muscular dystrophy (OPMD), X-linked mental retardation (XLMR), cleidocranial dysplasia (CCD), congenital central hypoventilation syndrome (CCHS), hand-foot-genital syndrome (HFGS), holoprosencephaly (HPE), sensory processing disorder (SPD), X-linked lissencephaly with abnormal genitalia (XLAG), and X-linked mental retardation with growth hormone deficiency (XLMRGHD).

The short tandem repeat (STR) expansions of DNA were discovered to cause hereditary diseases in the early 1990s and are currently known to be linked with more than 50 developmental, neurodegenerative, or neuromuscular diseases [1], including fragile X syndrome (FXS), several spinocerebellar ataxias (SCAs, including the most recently reported SCA50/ATX-FGF14 [2,3]), Huntington’s disease (HD), myotonic dystrophies types 1 and 2 (DM1 and DM2), and frontotemporal dementia/amyotrophic lateral sclerosis (FTD/ALS). Repeat expansion diseases are caused by unstable expanded tri-, tetra-, penta-, hexa- or dodecanucleotides that can be located in coding or noncoding gene regions, namely 5′-untranslated regions (UTR), 3′-UTR, introns, or promoters (Figure 1) [1]. In recent years, a new group of five trinucleotide repeat expansion diseases has been reported, including neuronal intranuclear inclusion disease (NIID) [4,5], oculopharyngeal myopathy with leukoencephalopathy 1 (OPML1) [4], and oculopharyngodistal myopathies 1–4 (OPDM1–4) [4,6,7,8,9]. Beyond the simple STR expansions, pentanucleotide pathological insertions have also been identified, with the first being the (TGGAA)_n_ in SCA31 [10] and later the (ATTTC)_n_ in SCA37 [11]. Since then, repeat insertions of (ATTTC)_n_ in six genes have been reported causing familial adult myoclonic epilepsies (FAME 1–4, 6, and 7) (Figure 1) [12,13,14,15,16,17].

Many of the known repeat expansions are transcribed bidirectionally from both DNA strands [18]. More recently, bidirectional transcription has been reported for OPML1-associated gene *LOC642361/NUTM2B-AS1* [4] and *RILPL1* in OPDM4 [9]. The vast diversity of genes containing disease-causing repeat expansions, combined with the rapid increase in the number of pathogenic STRs that have been discovered in the last years, highlights the need for studying the molecular mechanisms triggered by these mutations. For transcribed DNA repeat expansions, the RNA repeats lead to cellular pathology by a complex mechanism that includes formation of abnormal RNA and protein aggregates, which have been extensively reviewed [1,18,19,20]. Considering that the majority of the known pathogenic repeat expansions cause developmental or/and neurodegenerative phenotypes, this suggests they impair critical cellular processes for development and neuronal function. Interestingly, when carefully analyzing the function of genes with repeat expansions, it is evident that a large number encode proteins with transcription factor activity (Figure 1). Several of them have CAG repeats encoding polyglutamine (polyQ) tracts that, when expanded, cause SCAs, such as *ATXN1* (SCA1), α-1ACT cistron of *CACNA1A* (SCA6), *ATXN7* (SCA7), and *TBP* (SCA17), while others have (GCN)_n_ sequences encoding polyalanine (polyA), such as *FOXL2*, *RUNX2*, *HOXA13*, and *HOXD13*, which cause developmental syndromes when expanded. A considerable number of genes containing repeats encode important players in synaptic function such as the *FMR1* and *DAB1* genes, implicated in FXS/FXTAS and SCA37, respectively [1,11].

Considering that many repeat expansions have arisen in genes encoding proteins with a specific function in transcription and gene expression regulation, this raises the hypothesis of a key role for wide transcription dysregulation in these diseases. This review will focus on transcriptional dysregulation as one of the crucial mechanisms underlying many of the neurodegenerative and neuromuscular repeat expansion diseases.

## 2. A Brief Overview on DNA Transcription of Coding Genes

**Figure 2:**
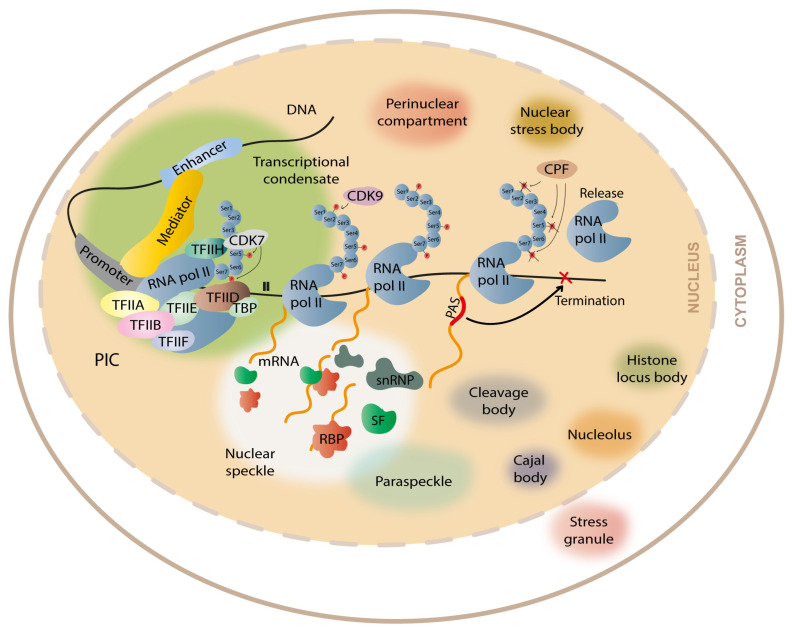
Representation of cellular condensates formed by liquid/liquid phase separation. The formation of transcriptional condensates occurs through the association of the intrinsically disordered regions of mediator, RNA pol II, and the transcription factors that form the pre-initiation complex (PIC), including transcription initiation factor IIA (TFIIA), TFIIB, TFIID (which is composed by TBP and the 14 TBP-associated factors), TFIIE, TFIIF, and TFIIH (which contains CDK7). The phosphorylation of the CTD-terminal of RNA pol II by CDK7 and CDK9 enables the RNA pol II to escape promoter-pausing and enroll transcription elongation. When RNA pol II transcribes the polyadenylation signal (PAS), being released from the DNA template, it produces a signal to terminate transcription. Closely associated with transcriptional condensates are the nuclear speckles, small reservoirs of splicing factors (SFs), RNA-binding proteins (RBPs), and small nuclear ribonucleoproteins (snRNP), and paraspeckles, all having a fundamental role in the mRNA processing. There are other membraneless organelles in the cell formed by LLPS, such as cleavage bodies, perinuclear compartments, Cajal bodies, nucleoli, histone locus bodies, nuclear stress bodies, and stress granules.

DNA transcription can be divided into four main steps: (1) initiation, (2) promoter-proximal pausing, (3) elongation, and (4) termination. RNA Polymerase II (RNA Pol II) is responsible for the transcription of protein-coding genes in eukaryotes. To enable the binding of RNA Pol II to the DNA and the transcription initiation, the pre-initiation complex (PIC) needs to be formed (Figure 2). PIC assembly occurs at the promoter and it includes RNA Pol II (composed by 12 subunits) and a variety of general transcription factors: transcription initiation factor IIA (TFIIA), TFIIB, TFIID (composed by TATA binding protein, abbreviated TBP, and 14 TBP-associated factors), TFIIE, TFIIF, and TFIIH, which contains the cyclin-dependent kinase 7 (CDK7) [21]. There is also an important complex, called the mediator complex, that facilitates the recruitment of the PIC components to the promoter and mediates PIC interaction with RNA Pol II [22]. If the promoter is methylated at CpGs, PIC assembly is compromised, and it leads to gene silencing, as occurs in CGG repeat expansion diseases such as FXS [23], or if the repeat expansion induces histone modifications typical of repressed genes, it can impair transcription initiation and/or elongation, as reported in FRDA [24]. On the other hand, if the promoter is unmethylated, PIC assembly succeeds. After PIC assembly, RNA Pol II initiates gene transcription at the transcription start site (TSS); however, to continue transcribing the RNA, the RNA Pol II needs to dissociate from the transcription initiation factors bound to the promoter in a process named promoter escape. This dissociation occurs when serine 5 and serine 7 of the carboxy-terminal domain (CTD) of RNA Pol II are phosphorylated by the CDK7 in TFIIH. The promoter escape allows RNA Pol II to produce a short nascent RNA. Then, the 5,6-dichloro-1-β-D-ribofuranosylbenzimidazole (DRB) sensitivity inducing factor (DSIF) and the negative elongation factor (NELF) bind to RNA Pol II leading to its pause a few nucleotides after the TSS (promoter-proximal pausing). The pause/release is mediated by the phosphorylation of serine 2 of the CTD domain of RNA Pol II, DSIF, and NELF, carried out by cyclin dependent kinase 9 (CDK9), which is a subunit of positive transcription elongation factor b (P-TEFb). This allows RNA Pol II to enroll in transcription elongation [25]. When the RNA is completely transcribed from the DNA template, transcription termination occurs. Briefly, transcription termination is dependent on polyadenylation signals (PAS) in the pre-mRNAs that mediate the cleavage and polyadenylation (CPA) of the nascent transcript [26]. During termination, the CTD of RNA Pol II is also dephosphorylated in the tyrosine 1 by the cleavage and polyadenylation factor (CPF) [27,28]. This dephosphorylation is crucial because the RNA Pol II can only join the PIC to reinitiate transcription if in the unphosphorylated form [29]. Depending on the complexity of the sequence to be transcribed, transcriptional steps can occur with more or less fluidity. In the case of repetitive sequences, RNA Pol II may face some troubles, either due to the formation of DNA tertiary/quaternary structures or due to the extension of the repetitive tracts. In fact, transcriptional abortion was reported in several repeat expansion diseases, such as FTD/ALS [30] and FAME1 [16]. The way that RNA Pol II has to face the repeat expansions is by interacting with DSIF and PAF1 complexes, which act on solving the DNA structures so that RNA Pol II can slide through the DNA molecule more efficiently [31].

As transcription is an incredibly dynamic process and tightly regulated, there is more than one RNA Pol II bound to the same DNA at different locations that initiate the transcription at different time points, which allows the increase in the number of mRNA molecules produced. Additionally, some mRNA processing events (e.g., splicing and polyadenylation) mostly occur during the elongation phase, while RNA Pol II slides through the DNA molecule. The pre-mRNA splicing, a process where the introns are removed and the exons are ligated to each other, occurs in the spliceosome after recognition of splice sites in the pre-mRNA. There is evidence that the catalytic core of the spliceosome is physically close to RNA Pol II, suggesting that transcription and splicing occur co-transcriptionally. Thus, the transcription and splicing machinery may be spatially organized, allowing their interaction [32]. Furthermore, the cleavage and polyadenylation factors were shown to interact with the CTD of RNA Pol II [33,34], suggesting that polyadenylation also occurs co-transcriptionally. In recent years, there is evidence that the efficient co-transcriptional processing of pre-mRNAs is possible due to the concentration of the transcription and processing machineries into subnuclear membraneless organelles formed by liquid-liquid phase separation (LLPS). The recent findings (1) that RNA Pol-II-mediated transcription occurs inside nuclear condensates [35,36]; (2) that an interaction between RNA Pol-II and splicing, cleavage, and polyadenylation factors exists; as well as (3) the recently described association between LLPS and polyadenylation in plants [37] give strength to that hypothesis.

## 3. Formation of Membraneless Organelles during Transcription and Gene Expression

It is well known that cells contain organelles delimitated by membranes (e.g., nucleus, Golgi complex). Beyond that, cells also contain several membraneless compartments formed by LLPS [38,39,40]. LLPS is a thermodynamically driven and reversible phenomenon that allows the condensation of macromolecules into liquid-like compartments that become separated from the diluted environment [39]. When the local concentration of macromolecules increases above a given threshold, dense liquid droplets enriched in macromolecules and RNA appear and are well separated from the dilute phase [41]. The formation of these compartments generates a unique environment that may favor the occurrence of several cellular processes, such as the assembly of the mitotic spindle during cell division [42], transcription [35,43,44,45,46], RNA metabolism [47], and stress response [48]. In the last years, LLPS has been thought to be responsible for the assembly of several membraneless organelles, including nucleoli, where rRNA is synthesized; P-bodies, where mRNA decay occur; nuclear speckles, which are reservoirs of RBPs acting on splicing; DNA repair centers, which concentrate DNA repair proteins; and stress granules, where certain RBPs concentrate in stress conditions to act on alternative splicing as well as on the formation of transcriptional condensates (Figure 2) [1,40,49,50,51,52,53].

Most of the proteins driving intracellular phase separation show conformational heterogeneity and have intrinsically disordered regions (IDRs) [54]. IDRs are very flexible regions that do not fold into globular three-dimensional structures and can exist in a variety of conformations [55]. Therefore, they can establish transient interactions with other proteins, allowing the establishment of networks with liquid-like properties. Although IDRs may play a vital role in LLPS, the interplay between globular domains and IDRs has been shown to be relevant for the assembly and recruitment of proteins for biomolecular condensates [56,57,58].

**Figure 3:**
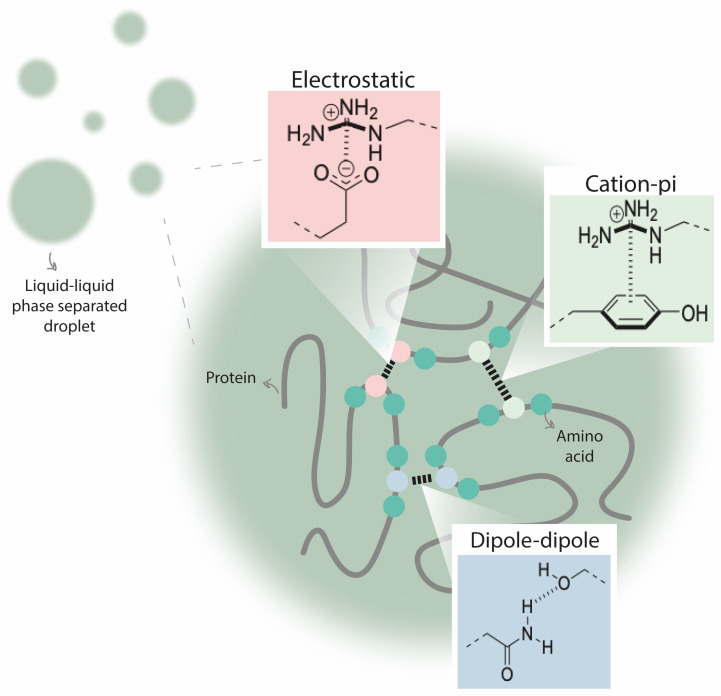
Different interactions between amino acids that are crucial driving forces for liquid/liquid phase separation (LLPS). Electrostatic interactions comprise the attractive or repulsive interactions that are established between charged molecules. Cation–pi interactions are noncovalent interactions between an electron-rich pi system (e.g., benzene) and a cation (e.g., Na^+^). Dipole–dipole interactions are attractive forces between the positive end of one polar molecule and the negative end of another polar molecule. Additionally, pi–pi interactions (established between two aromatic species) and hydrophobic interactions (repulsive forces between nonpolar molecules and water) are also important for driving LLPS.

There are several characteristics that help to define an IDR from the main structure of the protein: charge, hydrophobicity, flexibility, sequence complexity, and amino acid composition. For example, some IDRs are enriched in disorder-promoting amino acids (Ala, Arg, Gln, Glu, Gly, Lys, Pro, and Ser) and contain few order-promoting amino acids (Asn, Cys, Ile, Leu, Trp, Tyr, Phe, and Val) [59]. The interactions that promote phase separation include electrostatic, cation–pi, dipole–dipole, hydrophobic, or pi–pi interactions, and they are crucial driving forces for biomolecular condensate assembly (Figure 3) [60]. Although IDRs have numerous biophysical features that may be determinant for inducing LLPS, protein disorder by itself is not a main driver for protein phase separation [41,55].

Short repetitive motifs, such as polyQ or polyA repeats, are low complexity regions, usually polymorphic, that form blocks of equal types of interactions thereby increasing multivalency, a feature normally associated with the formation of biomolecular condensates [39,61]. Interestingly, a growing number of polyQ and polyA disease-associated proteins have recently been shown to be prone to form LLPS condensates [62,63,64,65]. In recent years, several studies have highlighted the sequence features needed for LLPS [58,66,67], and based on the biophysical properties of the amino acids in IDRs, multiple algorithms have been developed to predict disorder and LLPS propensities [68,69,70]. After a protein is predicted to be disordered or to drive LLPS, experimental approaches need to be performed to evaluate if the protein can undergo phase separation [71,72].

## 4. Repeat Expansions in Proteins Alter Their Condensation Behavior

Transcriptional condensates are composed by several proteins, such as transcription factors (TFs) and co-activators. Their formation contributes to approximate the transcriptional machinery, DNA template, and respective *cis*-regulatory elements. The formation of these concentrated dynamic compartments increases the proximity between components needed for transcriptional activation. Several TFs (e.g., OCT4 and SP1) have low complexity regions that allow them to phase separate and form discrete nuclear puncta in cells [36,73,74,75], and some studies have shown RNA Pol II colocalizing with TFs into puncta with liquid-like properties in live cells (Table 1) [36,51]. Furthermore, their transcriptional partners co-activator BRD4 [35] and mediator [75] form phase-separated compartments that recruit RNA Pol II to the transcriptional start site. Moreover, several RNA-binding proteins (RBPs), namely FUS, EWS, TAF15, hnRNPA1, TDP-43, and Matrin-3 can themselves undergo phase separation *in vivo* (Table 1) [40,47,76,77,78,79,80]. Notably, increasing evidence suggests that various RBPs may control transcription in an RNA-mediated manner, promoting enhancer/promoter looping, as in the case of YY1 functioning as TFs [81]. In fact, ChIP-seq has shown a large number of TFs and RBPs in promoters and enhancers, indicating that they may have a function at chromatin level [81,82]. These findings support the hypothesis that the formation of condensates can provide spatial possibilities for diverse local biochemical processes to take place simultaneously without perturbing each other, and, as the condensates are highly dynamic, the establishment of multiple interactions within and between condensates creates a rapid flux of molecules among them, allowing the co-occurrence of several RNA processing mechanisms (e.g., transcription elongation, splicing, and polyadenylation). Thus, the maintenance of the molecular features necessary for preserving the condensate dynamics is crucial for gene expression regulation. Interestingly, several proteins with repeat tracts encoding polyglutamine, such as ATXN1, ATXN2, ATXN3 and TBP, which, when expanded, cause neurodegenerative diseases, have been shown to form aberrant protein aggregates by LLPS [62,64,65,83]. In SCA1, both the ATXN1 with an expansion of 30 glutamines [30Q] and ATXN1-[85Q] can form nuclear bodies by LLPS that are converted in solid–gel aggregates under stress conditions, possibly being involved in SCA1 neurotoxicity [64]. As the proteins encoded by disease-associated genes in repeat expansion disorders have low complexity regions composed by polyglutamine expansions (e.g., ATXN7 in SCA7 and CACNA1A/α-1ACT in SCA6), they are also prone to misfold, as shown for the abovementioned proteins (Table 1). Remarkably, alterations in LLPS behavior have already been linked to several neurodegenerative diseases. In amyotrophic lateral sclerosis (ALS), disease-associated mutations are thought to alter the capacity of TDP-43 and FUS to participate in complexes mediated by phase separation, disrupting their normal function [84,85]. In frontotemporal dementia (FTD), mutations can alter the properties of TIA1 protein, a prominent stress granules component, increasing their capacity to phase separate and altering the stress granule dynamics [86]. In Alzheimer’s disease, disease-associated mutations alter the phase separation capacity of tau, leading to the formation of pathogenic aggregates [87]. Thus, they can theoretically promote the formation of aggregates that might be involved in the pathogenic mechanisms of these neurodegenerative diseases. However, as the amino acid composition and the presence of IDRs are not the only factor influencing the phase separation capacity, this assumption needs to be experimentally verified to understand if disease-associated LLPS alterations might underlie these and other neurological/neurodegenerative diseases.

As the IDRs of proteins are known to favor protein clustering and, thus, drive LLPS, alterations in these regions might affect the formation of condensates. Repeat expansions of low complexity regions can modify the conformational features of the protein, interfering with protein/protein interactions, altering the phase separation capacity of these proteins, and, consequently, disrupting the formation of condensates or changing their material properties. It has been recently reported that repeat expansions occurring in IDRs of several TFs alter their phase separation capacity and capacity to co-condense with the transcription machinery. Basu et al., 2020 [62] demonstrated this aberrant phase separation for proteins encoded by *HOXD13*, *HOXA13*, *RUNX2*, and *TBP*, which are genes with repeat expansions encoding polyA or polyQ, associated with synpolydactyly, hand-foot genital syndrome (HFGS), cleidocranial dysplasia (CCD), and spinocerebellar ataxia type 17 (SCA17), respectively. For the HOXD13 protein, alanine repeat expansions were shown to enhance the phase separation capacity of its IDR, resulting in a decreased capacity of this TF to co-condense with mediator, a phenomenon the authors named “condensate unblending” [62]. This condensate unblending resulted in changes of expression of HOXD13 target genes in several cell types. In HOXA13 and RUNX2, alanine repeat expansions were shown to increase the phase separation capacity of their IDR, leading also to a decrease of the co-condensation with mediator. For TBP, the disease-associated glutamine repeat expansion originated a decrease in the phase separation capacity of this TF [62]. Interestingly, it has been reported that the androgen receptor (AR), harboring a CAG repeat that when expanded causes spinal bulbar muscular atrophy (SBMA), has the ability to form phase separation condensates [90]. Curiously, the AR transcription factor interacts with MED1 by forming condensates where active transcription occurs. It is reported that point mutations in several AR domains inhibit the co-condensation with MED1, altering its transcriptional activity [90]. Thus, if the CAG repeat expansion goes beyond a given threshold, as occurs in SMBA, this could impair the AR/MED1 condensate formation and, consequently, lead to transcriptional dysregulation, although no evidence for this has been reported so far. Hence, aberrant alterations in phase separation properties of proteins with pathogenic repeat expansions and/or alterations in the co-condensation behavior with other important cellular components could be a pathogenic mechanism shared by diseases caused by repeat expansions in coding regions.

## 5. Transcriptional Dysregulation in Coding Repeat Expansion Diseases

There is evidence of wide transcriptional dysregulation in several repeat expansion diseases, which are often called transcriptionopathies. Several of the disease-associated genes harbor pathogenic repeats that, when expanded, may alter the transcription of that specific gene and/or cause expression alterations in their target genes due to abnormal interactions with important players in the transcriptional machinery (Table 2).

SCA1, the first SCA with the gene assigned to a chromosomal location, is caused by an expanded (CAG)_>40_ in the *ATXN1* gene, which results in the expression of a protein with an expanded polyglutamine [123]. Mutant ATXN1 (mATXN1) accumulates and aggregates in the nucleus of SCA1 human brain tissue, SCA1 mouse model, and HeLa transfected cells [124], impairing the function of the nonpathogenic ATXN1 protein. ATXN1 is a transcription factor that function as a repressor and interacts with several proteins with transcriptional regulatory roles, such as (1) histone deacetylase 3 (HDAC3), which deacetylate lysines of histone proteins resulting in transcriptional repression; (2) RoRα/TIP60 complex, which has transcription factor activity crucial for cerebellar development; (3) Ataxin-1-like protein (ATXN1L) or BOAT, which is a functionally redundant ATXN1 homolog; (4) Sp1, a transcription factor; (5) capicua (CIC), a transcriptional repressor; and (6) polyglutamine-binding protein 1 (PQBP1), involved in transcription activation [92,94,97,99,103]. The polyglutamine expansion affects the normal interactions of ATXN1 with these transcriptional regulators, resulting in transcriptional dysregulation of target genes, supporting the hypothesis that alterations in transcription can be one of the SCA1 pathogenic mechanisms.

One of the first mechanisms explaining the transcriptional alterations in SCA1 was based on the role of PQBP1, which is a binding partner of RNA pol II that activates transcription [94]. This protein binds to repetitive motifs at the C-terminal domain of RNA pol II, especially when serine 1 is phosphorylated (elongation phase) [94]. However, it also binds to the polyglutamine region of ATXN1 that, when expanded in mATXN1, increases not only the number of PQBP1 proteins that bind to the mATXN1 but also the affinity between the PQBP1 and phosphorylated RNA pol II [94]. The sequestration of PQBP1 by the mATXN1 aggregates, and the consequent sequestration of phosphorylated RNA pol II, leads to the decrease of the available phosphorylated RNA pol II necessary to elongate transcription, resulting in gene expression dysregulation. As shown by Cummings and colleagues (1998), mATXN1 accumulates in neuronal nuclei in human and mouse SCA1 brain tissue [124]. Thus, the formation of mATXN1/PQBP1/RNA pol II complexes causes transcription dysregulation, especially in brain-specific genes. Ingram and collaborators (2016) performed RNA-seq to create a profile of cerebellar gene expression in mouse models of SCA1, identifying several dysregulated genes that correlate with SCA1 progression [104]. Interestingly, huntingtin (HTT), the polyglutamine-expanded protein in HD, also binds to PQBP1 in its expanded polyglutamine form [125], so the same mechanism of impaired transcription might occur in HD, although no evidence for that impairment has been reported so far. All these proteins, ATXN1, PQBP1, RNA pol II, and HTT, have the capacity to form liquid-like compartments by phase separation [63,64,74,126], so the interaction of mATXN1 or mHTT with PQBP1 and RNA pol II modifies their physical properties and, consequently, their co-condensation behavior, leading to alterations in gene expression. More recently, Rousseaux and colleagues (2018) found that mATXN1 can cause cerebellar toxicity through its interaction with CIC [103]. The ATXN1/CIC complex is known to be important for development, functioning as a transcriptional repressor complex. The gain of function of this complex leads to neurodegeneration throughout the repression of important developmental genes, but its loss of function also results in hyperactivity, impaired learning and memory, and deficits in upper-layer cortical neuron activity, showing that its repressor activity in specific genes is important for several neuronal functions [127]. Rousseaux and colleagues (2018) have shown that the transcriptional changes seen in Purkinje cells of SCA1 patients did not occur when the mATXN1 was inhibited from binding CIC in neurons derived from SCA1 patients, implying that the mATXN1/CIC interaction is the mediator of these transcriptional changes. This suggests that the formation of mATXN1/CIC complex is crucial to trigger SCA1 disease.

As shown in Table 2, a variety of proteins with transcriptional regulation function interact directly with proteins with mutations associated with different types of SCAs. Similar to ATXN1, polyglutamine expansion at other proteins, e.g., TBP, ATXN7, might alter their interaction properties, leading to alterations in the expression of their target genes.

## 6. Aberrant Condensates and Transcriptional Dysregulation in Noncoding Repeat Expansion Diseases

The alteration of phase separation capacity by repeat expansions in coding gene regions is easily understandable. However, in noncoding gene regions, a question remains to be answered: How can a repeat expansion lead to alterations in phase separation capacity with consequent changes in gene transcription?

RNA molecules play important functions in the formation of different cellular condensates, such as the nucleolus, nuclear speckles, paraspeckles, and stress granules [128]. RNA can also be a strong regulator of transcriptional condensate dynamics as its high negative charge density, given by the phosphate backbone (proportional to their length), can easily alter the electrostatic interactions driving condensate assembly [39,129]. In fact, there is evidence that an appropriate amount of RNA can enhance condensate formation, while high RNA concentration dissolves them [130], suggesting that the RNA:protein ratio affects the phase separation process. Sharp and colleagues (2022) [131] identified a model where the RNA mediates a nonequilibrium feedback mechanism for transcription. At the beginning of transcription, there is a small proportion of RNA molecules being transcribed. As the RNA molecules are being transcribed during elongation, the high levels of RNA promote condensate dissolution. Interactions with nascent small RNAs could stimulate the rate of condensate assembly. The negative charges of small RNAs contribute to the formation of transcriptional condensates and the production of, for example, eRNAs at enhancers. This is important for controlling the frequency of transcriptional hubs and, consequently, controls the rate of synthesis of mRNA [131]. Thus, if the amount of RNA can regulate transcriptional condensate formation, it might also have the capacity to modulate the transcriptional profile as well. Thus, eRNAs transcribed bidirectionally at enhancers contribute to increase the frequency of transcriptional condensate formation, resulting in the upregulation of their target gene(s). Henninger et al. (2021) [132] showed that RNA can provide positive and negative feedback to transcription through the alterations of the electrostatic interactions required for transcriptional condensate formation. Transcriptional condensate assembly involves the crowding of TFs by the positive influence of eRNAs through electrostatic interactions and/or IDR/IDR interactions of TFs and co-activators [35,43]. Thus, as the eRNAs produced by enhancers have the capacity to stimulate condensate formation, they can also lead to transcriptional changes through alterations in mRNA production of specific genes.

Furthermore, the length and sequence of RNAs are also important for the regulation of condensate formation as these factors are crucial for the binding capacity of RBPs. While high concentrations of RNA can dissolve phase-separated compartments [130], the same does not happen with repetitive RNAs as they are able to form RNA secondary structures, creating the conditions to generate specific RNA/RNA and RNA/RBP interactions. One of the hallmarks of noncoding repeat expansion diseases is the accumulation of repeat-containing RNA transcripts into aberrant nuclear aggregates (RNA foci) [1]. These RNA aggregates co-localize with RBPs having transcription and/or splicing activity, such as hnRNPK in SCA10 transgenic mouse [133] and hnRNPA1 in C9Orf72-associated ALS patients, in cerebellar autopsy tissue [134]. Notably, both sense and antisense RNAs contribute to the formation of aberrant nuclear RNA aggregates in several repeat expansion diseases [18]. It was found that repetitive RNAs have the capacity to form nuclear aggregates by themselves [135]. The mechanisms by which they are generated are not yet completely understood; however, there is evidence that RNA can itself undergo phase-separation, without requiring proteins, when it has a repeat tract beyond a critical repeat number [135]. Jain and Vale (2017) demonstrated that several disease-associated repeat-containing RNAs, such as (CAG)_n_ in HD and SCAs (CUG)_n_ in myotonic dystrophy and (GGGGCC)_n_ in FTD/ALS, when expanded above a given threshold, can establish multivalent intermolecular base-pairing and electrostatic (requiring Mg^2+^) interactions, leading to the formation of nuclear puncta via LLPS or solid–gel transition [135]. The authors showed that the incorporation of antisense oligonucleotides (ASOs) or other agents that disrupt base-pairing or electrostatic interactions disrupt the formation of RNA foci *in vitro* proving that both interactions are essential for RNA foci formation. The importance of RNA secondary structures in phase separation has also been reported by Fay and collaborators (2017) [136], showing that a GGGGCC repeat RNA in a G-quadruplex conformation, containing four-stranded structures stabilized by guanidine tetrads connected by short loops, is able to drive the assembly of RNA granules composed by RNA and proteins formed by LLPS in the nucleus or cytoplasm. In fact, there is also evidence supporting that the ribonucleoprotein complexes, composed by RNA and RBPs, can form LLPS droplets *in vitro* [137,138,139].

However, it is not known to what extent the RNA aggregates are toxic by themselves. The neurotoxicity issues appear when phase-separated RNA aggregates sequester key proteins *in vivo*, impairing their normal functions. It is widely known that RNA foci can sequester RBPs, key regulators of RNA metabolism, such as mRNA splicing [140] and polyadenylation [141,142], and play a vital role in maintaining homeostasis in neuronal systems [143]. Thus, the aberrant nuclear RNA aggregation with RBP sequestration might impair the mRNA metabolism, as RBPs are no longer available to perform their specific roles, leading to splicing and polyadenylation misregulation, alterations in mRNA transport to the cytoplasm, or in translation. Splicing misregulation has been widely reported in noncoding repeat expansion diseases, such as in *C9Orf72*-FTD/ALS, in which RBP sequestration by RNA aggregates has been shown to be responsible for the formation of a variety of mRNA isoforms, completely changing the transcriptome in neuronal cells [144], while abnormal alternative polyadenylation has been reported for OPMD, in which the *PAPBN1* mutation leads to 3′-UTR shortening, and for FXS, caused by a repeat expansion in *FMR1* that affects the choice of polyA signals [145]. The impairment of the RNA processing machinery by aberrant RNA aggregation thus results in overall transcriptional alterations.

Considering that RBP sequestration, in abnormal phase-separated nuclear aggregates, is one of the major contributors to repeat expansion diseases and is identified in noncoding repeat disorders when transcription of the expanded repeat occurs [140], more efforts are needed to understand how to modulate this abnormal phase separation capacity for therapeutic purposes.

## 7. Conclusions and Future Perspectives

LLPS is crucial for many cellular processes such as the assembly of the transcriptional and mRNA processing machinery. It is becoming evident that changes in the propensity for local LLPS in specific cells or its precursors leads to aberrant RNA and protein aggregates, either nuclear or cytoplasmic, which are commonly associated with repeat expansion diseases. Alterations in protein:protein and protein:RNA interactions cause changes in condensate dynamics that consequently induce transcriptional and/or mRNA metabolism dysregulation. When these alterations occur in progenitor, neuronal, or muscle cells, they can lead to changes in the expression of cell-specific genes essential for developmental, brain, or muscle function.

To better understand aberrant condensate formation in repeat expansion diseases, the assessment of sense and antisense gene expression levels of disease-associated genes in affected cells and tissues from asymptomatic and affected subjects during development to aging is imperative. The use of autopsy material from affected individuals is important to explore disease outcomes. However, this material is difficult to access and only allows depiction of the end stage of the disease, which can be a result of cellular responses to the accumulation of toxic structures and not specifically the response to the repeat expansion itself. While this limitation hampers the advances in knowledge of the pathogenic mechanisms of repeat diseases, the use of animal models is crucial to better understand the molecular and cellular basis of disease and have provided an enormous contribution to this field.

In noncoding repeat expansion diseases, LLPS contribute to the formation of abnormal RNA aggregates with the sequestration of RBPs. The aberrant aggregation caused by alterations in the phase-separation behavior of these RNA:protein interactions results in an impairment of RBP function, which affects the pre-mRNA processing mechanisms, alters their binding to the transcriptional machinery or to the promoter itself, and inhibits their interaction with specific transcription factors. Altogether, this leads to a dysregulation of important players in transcription that can directly affect the formation of transcriptional condensates and alter the nuclear speckle conformation leading to mis-splicing and defective polyadenylation events, all resulting in a modification of the transcriptional profile.

As such, it is vital to focus on understanding the molecular processes underlying the formation of LLPS aberrant condensates present in affected cells of subjects with repeat expansion diseases because the propensity for phase separation of RNAs, proteins, and RBPs is likely affecting the expression of genes in these cells and is at the core of pathogenicity.

## Figures and Tables

**Table 1:** Proteins involved in neurodegenerative diseases with phase separation propensity.

Proteins with LLPS Capacity	UniProt ID	IDRs (n) ^a^	RRMs (n)	DBMs (n) ^a^	Disease	Experimental Model	References
FUS	P35637	1	1	1	ALS, FTD, SCA31	Human SK-ES-1 cells	[36,40,85]
EWS	Q01844	3	1	1	ALS, FTD, cancer		
TAF15	Q92804	3	1	1	ALS, FTD, cancer		
hnRNPA1	P09651	2	2	ND	ALS	Human HeLa and U2OS	[77,80]
TDP-43	Q13148	2	2	ND	ALS, SCA31	*In vitro*	[78,80,84]
MATRIN-3	P43243	4	2	1	ALS, FTD, distal myopathy	Yeast, mouse C2C12 myoblast cells	[88,89]
ATXN1	P54253	5	ND	ND	SCA1	Mouse Neuro 2a cells	[64]
ATXN2	Q99700	3	ND	ND	SCA2, ALS	Human U2OS cells	[83]
ATXN3	P54252	1	ND	ND	MJD/SCA3	*In vitro*	[65]
CACNA1A/α-1ACT	O00555	3	ND	^b^	SCA6	ND	
ATXN7	O15265	6	ND	ND	SCA7	ND	
TBP	P20226	3	ND	ND	SCA17	Human HEK293T cells	[62]
AR	P10275	2	ND	ND	SBMA	*In vitro* and in human LNCaP and LAPC4 cell lines	[90]

IDR: intrinsically disordered region; RRM: RNA recognition motif; DBM: DNA binding motif (zinc-finger domains); ND: not determined; ^a^ IDRs and DBMs predicted by MobiDB2; ^b^ No annotated DBM, but α-1ACT was shown to interact with DNA by Chip quantitative real-time PCR [91].

**Table 2:** Disease-associated expanded proteins interacting with transcriptional regulators.

Disease	Protein	Transcriptional Interactors	Dysregulated Genes	References
SCA1	ATXN1	HDAC3, RORα/TIP60, CIC, LANP, SMRT/NCOR2, HDAC4/MEF2, ATXNL1 (BOAT), GFI-1, 14-3-3, SP1, A1Up, PQBP1, RBM17	Cerebellar PC-specific	[92,93,94,95,96,97,98,99,100,101,102,103,104,105]
SCA2	ATXN2	PABP-1, A2BP1/Fox1, DDX6, TDP-43	Cerebellar GC- and PC-specific (e.g., *ROR*α, *Itpr1*, *Atp2a2*, *Inpp5a*)	[106]
MJD/SCA3	ATXN3	CBP, PCAF, P300, HDAC3/NCOR, FOXO4, TBP, PML, TAF130, MAML1, SC35, NCoR	Involved in glutamatergic neurotransmission; intracellular calcium signaling; MAP Kinase signaling	[107,108,109,110,111,112,113]
SCA6	CACNA1A	NA	Involved in neurite outgrowth (e.g., *TAF*, *BTG1*, *PMCA2*, *GRN*)	[91]
SCA7	ATXN7	GCN5, USP22, CRX, RORα, SIRT1, TBP, KAT2A/GCN5/ATXN7L3 (components of SAGA complex)	Cerebellar PC-specific; involved in morphological and physiological identities of mature photoreceptors	[114,115]
SCA17	TBP	TFIIB, XBP1, NFY, MYOD, SP1	*Hspb1*, *Manf*, *Trka*, *Mhc4*, *Mck*	[116,117,118,119,120,121,122]

PC—Purkinje cells; GC—granular cells; NA—not available.

## Data Availability

Not applicable.
